# Recurrent Conjunctival Melanoma Managed with Long-Term Eye-Preserving Treatment Followed by Delayed Eyelid Metastasis: A Case Report

**DOI:** 10.3390/jcm15093334

**Published:** 2026-04-27

**Authors:** Lidiya Zaduryan, Gabriela Vasileva, Elitsa Hristova, Mladena Radeva, Igor Resnick, Zornitsa Zlatarova

**Affiliations:** 1 University Specialized Eye Hospital, 9002 Varna, Bulgaria; 2 Department of Ophthalmology and Visual Sciences, Medical University of Varna, 9002 Varna, Bulgaria; 3 Department of Optometry and Occupational Diseases, Medical University of Varna, 9002 Varna, Bulgaria; 4 Department of Medical Genetics, Medical University of Varna, 9002 Varna, Bulgaria

**Keywords:** conjunctival melanoma, local recurrence, Mitomycin C, no-touch technique, eye preservation, locoregional metastasis, ocular surface melanoma

## Abstract

Background: Conjunctival melanoma is a rare but potentially aggressive ocular surface malignancy characterized by frequent local recurrence and risk of metastatic spread. In carefully selected cases, depending on tumor extent, clinical course, and patient condition, management may require balancing oncologic control with preservation of the globe, visual function, and quality of life. Case Presentation: We report the case of a 78-year-old woman with amelanotic conjunctival melanoma of the left eye. Initial treatment consisted of wide local excision using a no-touch technique, conjunctival autograft reconstruction, and adjuvant topical Mitomycin C (MMC). During a 10-year follow-up period, the patient developed multiple local recurrences requiring repeated surgical excisions and additional MMC therapy. Despite the chronic relapsing course, useful visual function and globe preservation were maintained. In December 2024, a subcutaneous lesion of the upper eyelid was detected and histopathologically confirmed as locoregional metastasis from the primary conjunctival melanoma. Given the patient’s advanced age, preserved visual function, absence of documented distant metastatic disease, and overall clinical context, management continued with a conservative, eye-preserving approach. Conclusions: This case illustrates that prolonged eye preservation may be achievable in carefully selected patients with recurrent conjunctival melanoma through repeated conservative management. However, this strategy does not eliminate the risk of delayed progression and requires individualized decision-making together with long-term surveillance.

## 1. Introduction

Conjunctival malignant melanoma (CMM) is a rare but potentially life-threatening ocular surface tumor arising from melanocytes within the conjunctival epithelium. Although it accounts for only a small proportion of all melanomas, its clinical significance is considerable due to its high propensity for local recurrence, multifocality, and metastatic spread [1,2,3].

CMM most frequently develops from primary acquired melanosis with atypia, but it may also arise from conjunctival nevi or de novo [3,4]. Clinically, it typically presents as a unilateral, elevated lesion that may be pigmented or amelanotic, often associated with prominent feeder vessels. The bulbar conjunctiva is the most common site of involvement, likely due to increased ultraviolet exposure. Importantly, the rich vascular and lymphatic network of the conjunctiva facilitates both local dissemination and distant metastasis. Reported recurrence rates range from 30% to 60% even after combined treatment [4,5,6]. Current management strategies include wide local excision using a “no-touch” surgical technique, combined with adjuvant therapies such as cryotherapy, topical chemotherapy (most commonly Mitomycin C), or radiotherapy. Despite these approaches, there is no universally accepted treatment algorithm, particularly in cases of recurrent disease. The rarity of the condition and the lack of large prospective studies further contribute to the absence of standardized guidelines [3,6].

Population-based studies from Sweden, the United States, and Europe have suggested increasing incidence trends and ongoing clinical importance of conjunctival melanoma over recent decades [7,8,9,10].

A central challenge in the management of recurrent conjunctival melanoma is the balance between achieving adequate oncologic control and preserving the eye. While radical procedures such as orbital exenteration may be considered in cases of extensive or repeatedly recurrent disease, they are associated with profound functional, cosmetic, and psychological consequences [4]. Conversely, repeated conservative, eye-preserving interventions may allow long-term local control and maintenance of vision but raise concerns regarding the potential for cumulative metastatic risk.

The present case is clinically unusual because it documents approximately annual recurrences over more than a decade, prolonged preservation of the globe through repeated conservative treatment, and subsequent delayed upper eyelid locoregional metastasis. Beyond its rare longitudinal course, the case highlights an important real-world therapeutic dilemma: how to balance oncologic risk, functional preservation, patient age, and quality of life when considering escalation to radical surgery. For these reasons, it offers relevant practical lessons for clinicians involved in ocular oncology care.

## 2. Case Presentation

**Figure 1:**
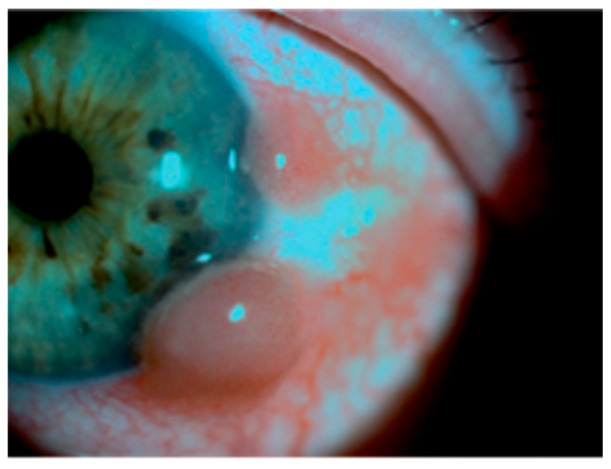
Slit-lamp photograph showing two amelanotic, vascularized nodular lesions on the temporal bulbar conjunctiva, measuring approximately 5–6 mm and 3–4 mm in diameter, with prominent feeder vessels and surrounding conjunctival hyperemia.

A 78-year-old woman presented in October 2013 with a progressively enlarging lesion of the left bulbar conjunctiva, associated with ocular irritation and excessive tearing over a two-month period (Figure 1). She reported a prior surgical excision of a similar conjunctival lesion at another institution before referral to our center; however, no external medical records or histopathologic documentation from that procedure were available. Therefore, the October 2013 visit represents the first fully documented presentation and treatment in our institution.

On examination, best-corrected visual acuity was 0.4 in the right eye and 0.6 in the left eye, with intraocular pressure measuring 18.5 mmHg bilaterally. Slit-lamp biomicroscopy revealed two amelanotic, yellowish-pink, vascularized nodular lesions located on the temporal bulbar conjunctiva, measuring approximately 5–6 mm and 3–4 mm in diameter, surrounded by conjunctival hyperemia. Both eyes were pseudophakic, and fundoscopic examination demonstrated angioretinal sclerotic changes.

Wide local excision with 2–3 mm safety margins was performed using a “no-touch” surgical technique, followed by conjunctival autograft reconstruction. The postoperative course was uneventful.

**Figure 2:**
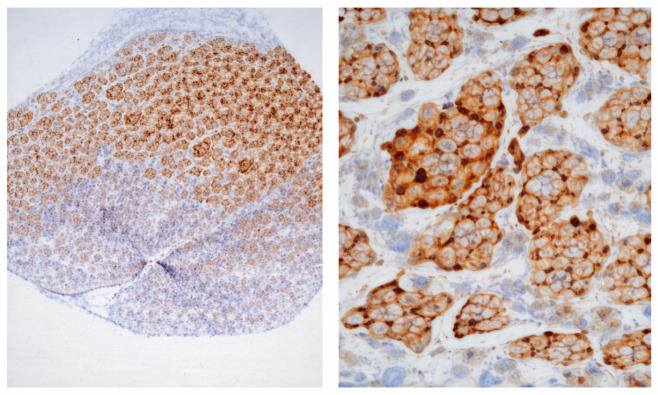
Histopathologic section of conjunctival tissue showing diffuse nests and cords of atypical melanocytes with large hyperchromatic vesicular nuclei and intracellular pigment granules. Pagetoid spread of malignant cells into the adjacent epithelium is evident, accompanied by a peritumoral lymphocytic infiltrate (HMB-45 staining: ×40, ×400).

Histopathological examination of the original conjunctival lesion was supported by immunohistochemistry demonstrating strong cytoplasmic expression of HMB-45 and Melan-A in tumor cells, consistent with amelanotic malignant melanoma. Subsequent recurrent lesions showed diffuse infiltration by atypical polymorphic melanocytic cells with hyperchromatic nuclei, prominent nucleoli, abundant eosinophilic cytoplasm, multinucleated tumor giant cells, and frequent mitotic activity, in keeping with recurrent melanoma. Focal pigmentation and a mild lymphoid stromal response with occasional intratumoral lymphocytes were also noted in later specimens (Figure 2).

Margin status, lymphovascular invasion, PAM-associated changes, and exact depth of invasion were not consistently reported in the archived pathology records.

Based on the available clinical and histopathologic records, the lesion was classified as de novo conjunctival melanoma, with no documented evidence of pre-existing primary acquired melanosis or nevus-related transformation.

Adjuvant topical Mitomycin C (MMC) 0.04% was administered in three cycles (four daily instillations for one week, alternating with treatment-free intervals). No clinically significant ocular surface toxicity related to MMC exposure was documented during follow-up.

**Figure 3:**
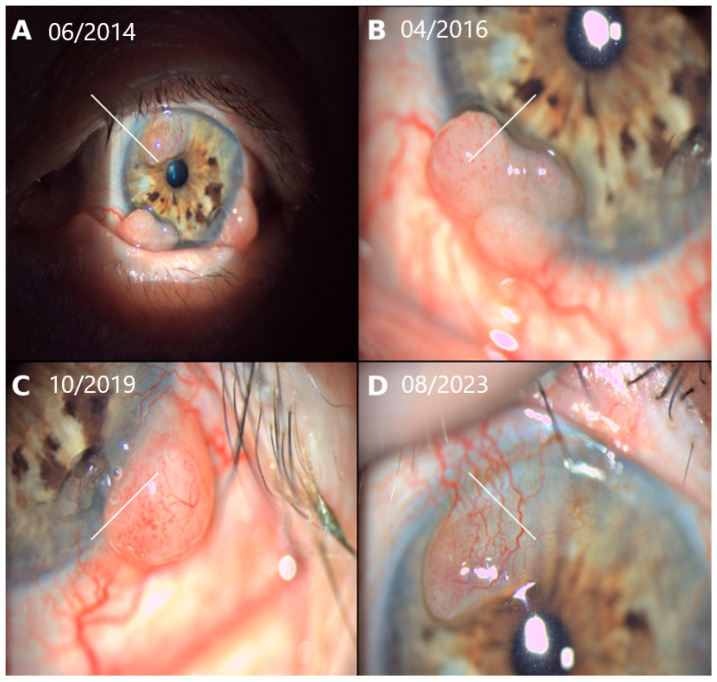
Serial slit-lamp images illustrating recurrent conjunctival melanoma over a 10-year follow-up period. (**A**) June 2014, (**B**) April 2016, (**C**) October 2019, and (**D**) August 2023. Images demonstrate recurrent amelanotic, vascularized nodular lesions arising within the previous surgical area. White line indicate sites of tumor recurrence, highlighting the chronic and persistent nature of the disease despite repeated excision and adjuvant therapy.

**Figure 4:**
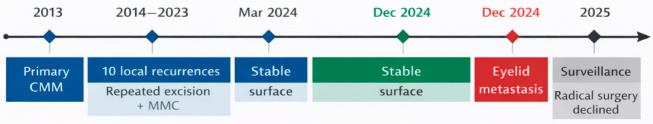
Graphical timeline summarizing the long-term clinical course of conjunctival malignant melanoma from 2013 to 2025. Following the initial diagnosis and primary surgical treatment in 2013, the patient developed recurrent disease with approximately annual local recurrences between 2014 and 2023, resulting in ten additional surgical excisions combined with adjuvant topical Mitomycin C. Clinical stability without visible recurrence was documented in March 2024. In December 2024, a locoregional metastatic lesion involving the upper eyelid was identified and locally excised. Ongoing surveillance continued thereafter, while radical surgery was declined.

Over a 10-year follow-up period, the patient developed multiple local recurrences requiring ten additional surgical excisions, each followed by adjuvant MMC therapy (Figure 3). The most recent surgically treated conjunctival recurrence occurred in August 2023, following the longest recurrence-free interval of the disease course since the previous intervention in September 2020. At that time, best-corrected visual acuity of the left eye was 0.3. Throughout this period, the patient was regularly followed in an oncology center and underwent periodic systemic surveillance, including laboratory evaluation with hepatic parameters and lactate dehydrogenase (LDH), as well as serial PET/CT imaging, with no evidence of distant metastatic disease. The prolonged clinical course and all major interventions are summarized in Figure 4.

**Figure 5:**
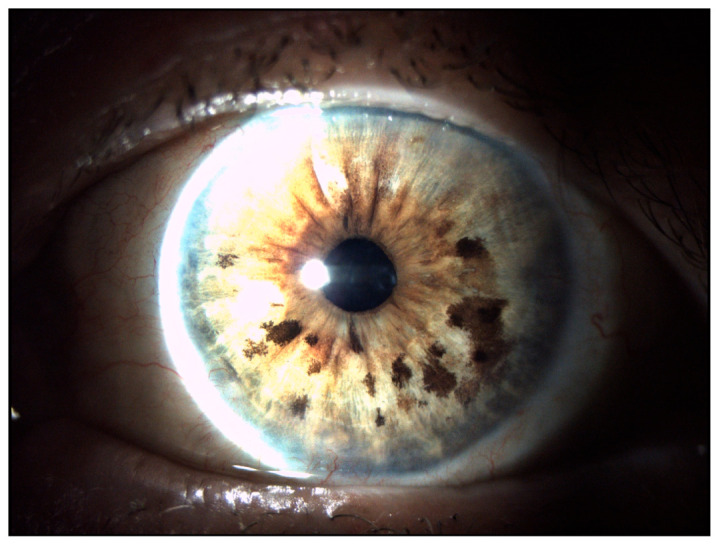
Slit-lamp photograph at the latest pre-metastatic follow-up (March 2024), demonstrating a stable ocular surface with no evidence of local recurrence. The bulbar conjunctiva appears quiet, with absence of nodular lesions or abnormal vascular proliferation.

At the latest pre-metastatic evaluation in March 2024, the ocular surface remained clinically stable, with no evidence of local recurrence. Best-corrected visual acuity of the left eye was 0.5 (Figure 5). No clinical or radiological evidence of regional or distant metastasis had been detected during follow-up.

**Figure 6:**
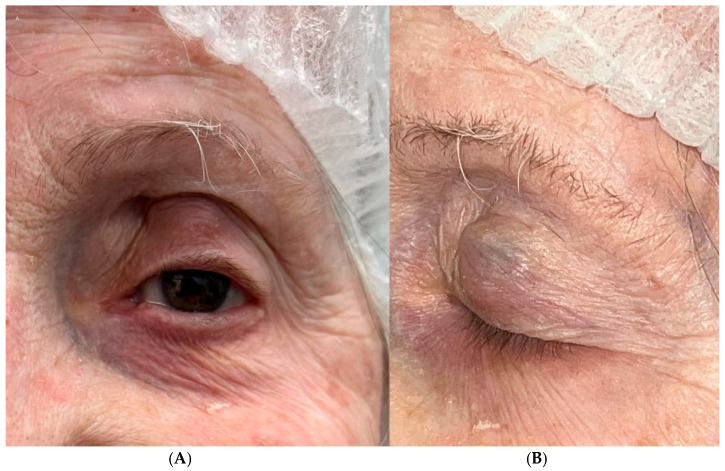
Clinical presentation of locoregional metastatic melanoma involving the upper eyelid of the left eye. (**A**) Frontal view demonstrating eyelid swelling and lesion prominence. (**B**) Lateral view showing a well-circumscribed, elevated subcutaneous mass with preserved overlying skin.

On a subsequent visit on 18 December 2024, a new subcutaneous, immobile, round lesion with firm consistency was identified in the upper eyelid of the left eye. Associated pigmentation of the tarsal conjunctiva was also observed. Local excision was performed, and histopathological examination confirmed metastatic melanoma consistent with the known conjunctival primary tumor (Figure 6).

Following confirmation of the eyelid metastasis, further systemic reassessment was recommended. However, due to the patient’s severely impaired general condition, no additional advanced imaging was performed. No escalation to radical surgery was undertaken, in accordance with the patient’s preference and overall clinical circumstances. At follow-up after excision of the eyelid lesion, best-corrected visual acuity of the left eye remained 0.5.

## 3. Discussion

### 3.1. Recurrence as a Chronic Feature of Conjunctival Melanoma

Conjunctival malignant melanoma (CMM) is notable for frequent local recurrence despite treatment, with reported rates ranging from 30% to over 60% [4,6,11]. The present case, marked by approximately annual recurrences over a 10-year period, supports the concept that CMM may behave as a chronic ocular surface disease with persistent biological activity rather than as a single isolated lesion, although in the present patient the lesion was clinically classified as de novo disease [11,12].

### 3.2. Late Locoregional Progression After Prolonged Local Control

A particularly important aspect of this case is the occurrence of upper eyelid locoregional metastasis after more than a decade of globe preservation and repeated local treatment. This illustrates that durable local control may coexist with persistent long-term progression risk.

Surveillance in CMM should therefore remain long-term and individualized rather than being discontinued after temporary disease quiescence. Cases combining prolonged eye preservation, repeated annual recurrences, and delayed upper eyelid metastasis appear uncommon, adding educational value to the present report.

In selected patients with conjunctival melanoma, surveillance may also require broader systemic assessment, as occult metastatic involvement can remain clinically silent. Depending on symptoms and clinical context, multimodal imaging—including echocardiographic evaluation when cardiac involvement is suspected—may provide additional diagnostic value in selected cases [13].

### 3.3. Biological Considerations and Future Perspectives

Conjunctival melanoma shares molecular features with cutaneous melanoma, including alterations involving the MAPK pathway [14,15,16,17]. This has created opportunities for targeted and immunotherapeutic strategies, which may become increasingly relevant in recurrent or advanced disease.

Although systemic therapy was not used in the present case, expanding medical treatment options may help reduce the need for disfiguring surgery in selected patients.

### 3.4. Practical Limits of Repeated Local Treatment

Wide local excision with adjuvant therapy remains a standard approach in recurrent conjunctival melanoma [18,19,20,21]. In the present patient, repeated excision combined with Mitomycin C enabled prolonged ocular preservation for many years.

Mitomycin C may help control residual microscopic or diffuse conjunctival disease [18]. In the present case, repeated treatment was clinically well tolerated, with no documented severe ocular surface toxicity such as limbal stem cell deficiency, persistent epithelial defects, or significant cicatricial change. However, the long-term course also demonstrates the limits of conservative treatment: recurrent visible lesions may be successfully managed, while the underlying capacity for locoregional or metastatic progression may persist. Repeated local therapy should therefore be regarded as long-term disease control rather than definitive cure in selected recurrent cases.

### 3.5. Decision-Making Beyond Oncologic Endpoints

Orbital exenteration is traditionally considered in advanced or repeatedly recurrent disease, although its survival benefit remains uncertain and its functional and psychological burden is substantial [22]. In the present patient, advanced age, preserved useful vision, absence of orbital invasion, lack of distant metastatic disease during prolonged follow-up, and patient preference all contributed to the decision to avoid radical surgery.

This case illustrates that treatment escalation should not rely solely on recurrence history. In selected elderly patients, management may reasonably incorporate functional outcomes, expected treatment burden, and quality of life alongside oncologic considerations.

### 3.6. Clinical Implications

This case supports an individualized approach to recurrent conjunctival melanoma. Repeated conservative treatment may preserve the eye for many years in carefully selected patients; however, such management requires lifelong surveillance and should be understood as disease control rather than elimination of future progression risk.

As a single case report, the present findings should be interpreted as hypothesis-generating rather than practice-defining.

## 4. Conclusions

Recurrent conjunctival melanoma represents a complex clinical challenge requiring a careful balance between oncologic safety and preservation of the eye. This case illustrates that prolonged local control and functional eye preservation may be achievable in carefully selected patients through repeated conservative management. However, such an approach does not eliminate the risk of delayed locoregional progression and requires individualized decision-making together with long-term surveillance. Further studies are needed to better define the relationship between local recurrence, treatment strategy, and metastatic risk.

## Data Availability

The original contributions presented in this study are included in the article. Further inquiries can be directed to the corresponding author(s).
